# Vitamin D, Insulin-Like Growth Factor, and Cognitive Performance: Age and Sex Variations

**DOI:** 10.1093/geroni/igab046.2618

**Published:** 2021-12-17

**Authors:** Magdalena Tolea, Stephanie Chrisphonte, James Galvin

**Affiliations:** 1 University of Miami Miller School of Medicine, Boca Raton, Florida, United States; 2 University of Miami Miller School of Medicine, Miami, Florida, United States

## Abstract

Vitamin D has been consistently linked to better cognitive function in observational studies. This impact may be due in part through its influence on neurotrophins. Whether the relationships between vitamin D, neurotrophins, and cognition vary based on biological factors such as age and sex is unclear. Using data from a sample of 400 community-dwelling older (mean age=75.3±9.4; 47% female) participants in a cross-sectional study of cognitive aging, we assessed relationships between plasma 25-hydroxy-Vitamin D and performance on a neuropsychological battery modeled after the UDSv3.0. Moderation by age and sex and the impact of vitamin D on the relationship between Insulin-like Growth Factor-1 and cognitive performance were assessed by linear regression stratified by sex and age (median split at 76y). We found vitamin D to be positively linked to global cognition (MoCA: β=0.095±0.025SE, p<0.001), working memory (Number Span Forward: β=0.017±0.007SE, p=0.011; Number Span Backward: β=0.016±0.007SE, p=0.028), episodic memory (Immediate Recall : β=0.089±0.027SE, p=0.001; Delayed Recall: β=0.047±0.015SE, p=0.002), attention and processing speed (Trail Making A: β=-0.365±0.163SE, p=0.026), executive function (Trail Making B: β=-0.537±0.215SE, p=0.014; Number-Symbol Coding: β=0.139±0.057SE, p=0.016), and an overall measure of cognitive function (z score: β=0.049±0.018SE, p=0.007). Most of these relationships were observed in women and younger older individuals (<76y). In addition, vitamin D increased the effect of IGF-1 on global cognition and memory by 13% and 8%, respectively. Our findings suggest that vitamin D-focused dementia prevention efforts would benefit if targeted to women and younger segments of the senior population and/or as an adjuvant to cognitive enhancement interventions.

